# Anti-rheumatic effect of quercetin and recent developments in nano formulation

**DOI:** 10.1039/d0ra08817j

**Published:** 2021-02-11

**Authors:** Feng Guan, Qi Wang, Yongping Bao, Yimin Chao

**Affiliations:** School of Pharmacy, Heilongjiang University of Chinese Medicine Harbin 150040 P. R. China guanfeng@hljucm.net; Norwich Medical School, University of East Anglia Norwich NR4 7UQ UK q.wang1@uea.ac.uk y.bao@uea.ac.uk; School of Chemistry, University of East Anglia Norwich NR4 7TJ UK y.chao@uea.ac.uk

## Abstract

Rheumatoid arthritis (RA) is a common worldwide chronic autoimmune disease, characterised by synovial hyperplasia, inflammatory cell infiltration, pannus formation and destruction of articular cartilage and bone matrix. It is one of the most common forms of osteoarthritis bestowing high rates of both disability and death. Increasing attention has been paid to the use of natural medicines and natural products in the treatment of RA and patients' acceptance has increased year by year because of their high efficacy and safety. Flavonoids are a group of important secondary metabolites occurring in many plants which have rich biological activities such as anti-rheumatic, vasodilator, and anti-tumor effects. Many successful medical treatments of RA appear to be attributable to the application of flavonoids. Quercetin, a representative active member of the flavonoid family, is found abundantly in many plants, *e.g.* apples, berries, cabbages, onions, and ginkgo. In recent years, progress has been made in the research of its anti-rheumatoid effects which indicate that it is potentially a noteworthy prodrug for the treatment of RA. However, the poor solubility of quercetin affects its bioavailability and clinical efficacy. This review aims to provide an up to date summary of the biological effects and mechanism of action of quercetin for the treatment of RA, and the research progress made towards nano formulations of quercetin to improve its solubility and efficacy.

## Abstract 2

Quercetin is a potential anti-rheumatoid drug. Nano formulation strategies could improve its solubility and efficacy.

## Graphical abstract

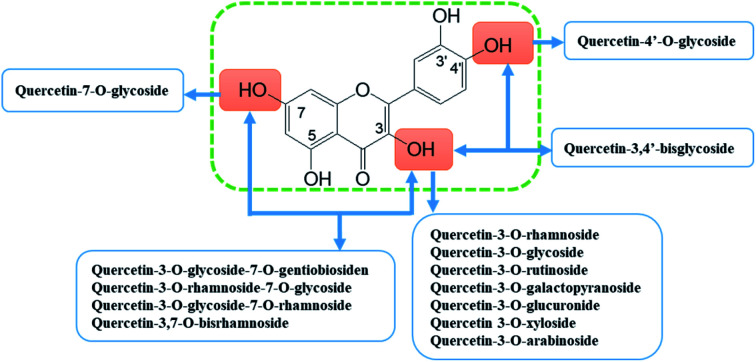

## 1 Introduction

Rheumatoid arthritis (RA) is a chronic autoimmune disease, characterised by synovial hyperplasia, inflammatory cell infiltration, pannus formation and destruction of articular cartilage and bone matrix.^1^ It is one of the most common and disabling forms of osteoarthritis. It is mainly manifested by redness, swelling, a hot sensation, pain, and other symptoms of the small joints of the extremities. The lesions develop symmetrically and destructively, which may eventually lead to joint deformity and loss of function, and can even affect the heart, lungs and nervous system.^2,3^ In most developed countries, RA affects 0.3–1.0% of the adult population.^4,5^ At present, steroidal, non-steroidal anti-inflammatory drugs (NSAIDs), disease modifying anti-rheumatic drugs (DMARDs), glucocorticoids, bacterial therapy^6^ and targeted treatment^7^ are used to relieve pain and control the disease. Patients with severe joint involvement may suffer disability, and may even require joint repair or replacement.^8,9^

However, long-term administration of these drugs may cause gastrointestinal discomfort, nausea, vomiting, and bleeding, or other adverse reactions such as to the central nervous system or cardiovascular system. Moreover, improper application of hormones may even aggravate the disease. When the disease is difficult to control, biological agents such as tumour necrosis factor inhibitors, abatacept, rituximab, and tocilizumab are often used in clinic. However their use is limited by high cost and adverse events *i.e.* reactions and infections at infusion and injection sites.^4^ Increasing attention has been paid to the use of natural medicines or natural products in the treatment of RA, and the patients' acceptance has increased year by year because of their high efficacy and safety.

**Fig. 1:**
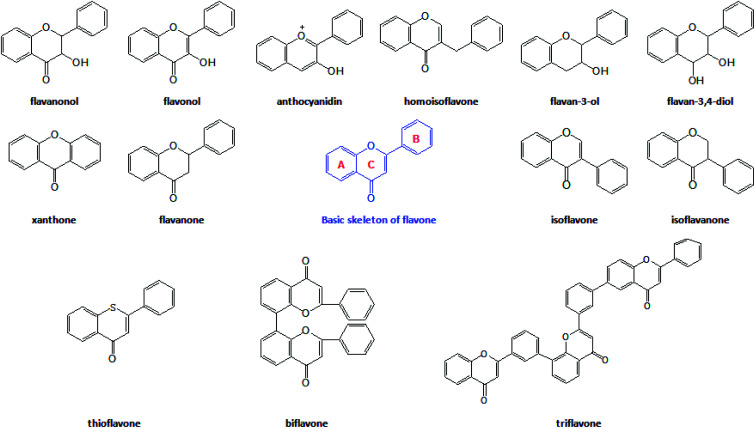
The basic structure and main types of flavonoids.

**Fig. 2:**
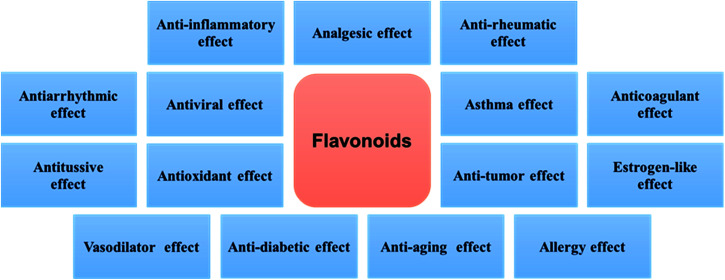
The main biological activities of flavonoids.

The flavonoids are vital secondary metabolites of many plants with the basic structural skeleton of 2-phenyl chromogenic ketone and consist of C6–C3–C6. It is a polyphenolic compound comprising two aromatic rings (A and B) and a heterocyclic ring (C). There are some flavonoid compounds that have a three-carbon chain but without a ring C. Some flavonoids exist in the form of dimers, trimers and even thioflavones. The main classes of natural flavonoids are flavones, isoflavones, flavanols, dihydroflavones, dihydroflavanols, bioflavonoids, triflavonoids, thioflavones, *etc.* (Fig. 1). Flavonoids have a number of biological activities (Fig. 2) including anti-inflammatory, analgesic, anti-rheumatic, vasodilator, anti-aging, and anti-tumour effects.^10,11^ The success of many medical treatments can be attributed to the application of flavonoids and new scientific studies of these compounds and their derivatives have focused on the activities above.^12,13^

**Fig. 3:**
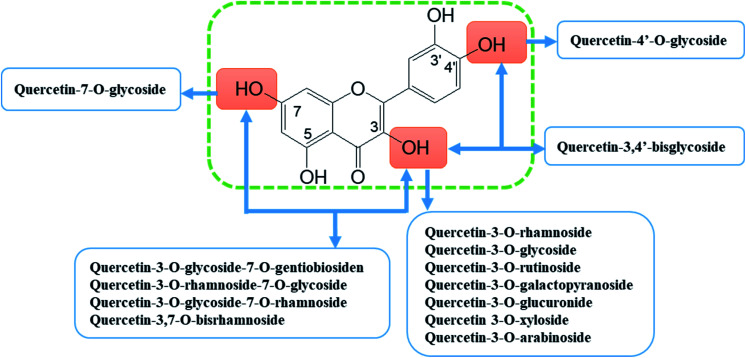
Quercetin and its main glycoside derivatives.

Quercetin (5,7,3′,4′-tetrahydroxyflavonol, C_15_H_10_O_7_) is a representative member of the flavonoid's family. It is found abundantly in a variety of foods including apples, berries, Brassica vegetables, grapes, onions, shallots, as well as many medicinal plants including Ginkgo biloba, Hypericum perforatum, and Sambucus canadensis.^14–16^ Quercetin frequently occurs as quercetin glycosides where polyhydroxyl substitution appears in its structure.^17^ The quercetin glycosides derivatives that have been identified include quercetin-3-*O*-rhamnoside (quercitrin), quercetin-3-*O*-glycoside (isoquercitrin), quercetin-3-*O*-rutinoside (rutin), and quercetin-7-*O*-glycoside (quercimeritrin) (Fig. 3).^18–20^ Quercetin possesses the typical pharmacological effects of flavonoids, such as anti-inflammatory, analgesic, anti-rheumatic, antioxidant, anti-tumour, *etc.*^12,21–24^. In recent years, new progress has been made in the research of its anti-rheumatoid effects which indicate that it is safe to use, with few side effects and thus a noteworthy prodrug for the treatment of RA.

Despite showing promising potential for medicinal use, the real-life application of quercetin has been largely limited due to its poor solubility and bioavailability.^13,18^ The solubility of quercetin in water is 7 μg mL^−1^, and it is absorbed and metabolised rapidly after entering the body. Quercetin has strong first pass effect and its bioavailability is very low, less than 3.6%. Quercetin is inactivated by combining with sugar molecules into gluconic acid and so on. Therefore, there is a need to employ modern nanotechnology to improve its solubility and bioavailability, so as to better deploy its anti-rheumatoid effects. This review is intended to provide an insight into the pharmacological function and mechanisms of action of therapeutic use of quercetin for RA. The recent research progress using nano formulation as a strategy to increase/improve quercetin potential in RA treatment have also been summarised. Our review is mainly based on the published journal papers in recent 15 years, excluding patent literature.

## 2 Anti-rheumatic effects of quercetin

The pathogenesis of RA is complex and has not been fully determined.^25^ Clinical treatment is mainly based on the purpose of reducing inflammation and alleviating symptoms. There is considerable research interest in the potential health benefits of quercetin. Therefore, the anti-rheumatoid effect of quercetin is summarised from the perspective of anti-inflammatory effects, analgesic effects, and the effect on experimental rheumatoid animal models.

### 2.1 Anti-inflammatory effect and mechanism of action

Inflammation plays a key role in rheumatoid diseases.^26^ Research results using both *in vitro* and animal models have shown that quercetin can inhibit the occurrence and development of inflammation, thus having an important potential impact on RA.

Liao and Lin studied the pharmacological effects of quercetin on systemic inflammation in septic mice.^27^ The sepsis mouse model was established by intraperitoneally (i.p.) injecting lipopolysaccharide (LPS). LPS is an important trigger of inflammatory response, which can stimulate a variety of cells *in vivo*, especially macrophages to synthesize and release many endogenous bioactive factors, leading to inflammatory response. They then administered quercetin to the septic mice in a prophylactic or therapeutic manner. Their results suggested that quercetin administration i.p. at a high dose of 0.15 μmol to each mouse could significantly (*P* < 0.05) increase Interleukin 10 (IL-10) secretion and had strong anti-inflammatory potential. Huang *et al.* similarly observed the anti-inflammatory effect of quercetin.^28^ Their results showed that quercetin at 100, 200, and 400 mg kg^−1^ could significantly inhibit the auricle swelling of rats caused by xylol, and the degree of swelling and inhibition rate were significantly different from those in the control group (*P* < 0.01). This suggests that quercetin can have a good inhibitory effect on inflammatory response.

RAW264.7 is a monocyte/macrophage-like cell line that has been frequently used to study immune function. Zhou *et al.* proved that quercetin significantly inhibited the increase of nitric oxide (NO), Tumour Necrosis Factor alpha (TNF-α), Interleukin 18 (IL-18) and Interleukin 6 (IL-6) in Raw264.7 cells induced by LPS (*P* < 0.01) showing that it has a good anti-inflammatory effect *in vitro*.^29^ These results agreed with the findings from Paul *et al.*^30^ and Cessak *et al.*^31^ which suggested that quercetin is an effective inhibitor for TNF-α and IL-6. Ren *et al.* also studied the protective effect of quercetin on LPS induced inflammation in RAW264.7 cells.^32^ Their results suggested that the protective effect may be related to the regulation of Toll-like receptor 4/nuclear factor kappa-light-chain-enhancer of activated B (TLR4/NF-κB) signalling pathway.

Chronic inflammation, a process linked to increased oxidative stress, may induce many diseases. Yeh *et al.* investigated the effects of β-carotene on the inflammatory reaction of macrophage model cells (differentiated HL-60 cells and RAW264.7 cells) and their modulation by quercetin or naringenin.^33^ Their results demonstrated that quercetin partially suppressed the pro-inflammatory effects, synergistically enhanced the inhibitory effects of β-carotene on the secretion of pro-inflammatory mediators and the DNA damaging ability of PMA-stimulated HL-60 cells. The mechanism of action was associated with its antioxidant activity and inhibition of the production of pro-inflammatory cytokines. Avila *et al.* summarised that quercetin showed a mixed inhibition mechanism towards Adenosine triphosphate (ATP) and that the binding site of quercetin overlaps with both ATP and inhibitor of nuclear factor kappa B (IκBα) binding sites.^34^

Extracellular High-Mobility Group Box-1 (HMGB-1) is an important late-stage inflammatory transmitter with strong inflammatory activity. It contributes to the pathogenesis of numerous chronic inflammatory and autoimmune diseases including RA. Musumeci *et al.* indicated that quercetin was a HMGB1 inhibitor and it could limit the activation of mitogen-activated protein kinase.^35^

### 2.2 Analgesic effect and mechanism of action

In addition to its significant anti-inflammatory effect, quercetin also shows significant analgesic activity.

Liu DN has reported that quercetin had no significant effect on paw withdrawal thermal latency in naïve rats. However, it could significantly increase the threshold of mechanical paw contraction response.^36^ The study also showed that quercetin has a significant inhibitory effect on bee venom induced spontaneous nociceptive response, pain score, thermal and mechanical pain sensitivity, and also on bee venom induced local inflammatory response.^36^ This suggests that quercetin's analgesic effect may be related to the blocking of pro-inflammatory factors. In addition, quercetin has a considerable inhibitory effect on the ipsilateral mechanical hyperalgesia and contralateral mechanical hyperalgesia caused by sciatic nerve branch injury model.

In addition to the above studies on the anti-inflammatory and analgesic effects of quercetin monomer, there are also a number related studies of medicinal plant extracts with quercetin as the main component, which further confirm the anti-inflammatory and analgesic effects of quercetin.^37–47^

### 2.3 Effects on experimental animal model

There is growing interest in the anti-rheumatoid effects of quercetin. The commonly used animal models of RA mainly include adjuvant induced arthritis (AA), collagen induced arthritis (CIA), oil induced arthritis (OIA), and proteoglycan induced arthritis (PGIA).^48–51^ In recent years, the research of anti-rheumatoid effect of quercetin has been mainly based on AA and CIA models.

#### 2.3.1 Effects on AA model

Guardia *et al.* studied anti-inflammatory effects of quercetin on adjuvant arthritis in rat using the Mizushima *et al.* model of acute and chronic inflammation.^52^ The results showed that quercetin could decrease the edema produced in the acute phase, induced 3–5 h after carrageenan injection (day 6). The reduction of paw volume was from 53% to 47%. However, subsequently in the chronic phase (day 7–30), quercetin had no significant effects.

Mamani-Matsuda *et al.* observed that the therapeutic and preventive properties of quercetin in experimental arthritis correlated with decreased macrophage inflammatory mediators.^53^ Their results indicated that in chronic rat (AA), oral administration of quercetin (30 mg per rat every 2 days, for 10 days) to arthritic rats resulted in a clear decrease of clinical signs compared to untreated controls. The effects of oral administration of quercetin (150 mg kg^−1^ daily, 28 days) were also investigated in a rat model of adjuvant arthritis by Gardi *et al.*^54^. Their results indicated that quercetin lowered levels of Interleukin 1β (IL-1β) (*p* < 0.003), monocyte chemotactic protein-1 (MCP-1) (*p* < 0.014) and restored plasma antioxidant capacity.

#### 2.3.2 Effects on CIA model

The anti-inflammatory and joint-protective properties of quercetin were studied using a C57BL/6 CIA model by Haleagrahara *et al.*^55^. This study determined that quercetin, which was non-toxic, produced better results than methotrexate for the protection of joints from arthritic inflammation in mice. Yang *et al.* also reported on the anti-rheumatoid effect of quercetin based on a CIA model.^56^ The results showed that quercetin could attenuate the CIA model *via* modulating the T follicular helper 17/regulatory T (Th17/Treg) balance, inhibiting NOD-like receptor protein 3 (NLRP3) inflammasome activation as well as activating Heme oxygenase-1 (HO-1)-mediated anti-inflammatory response. Wang *et al.* established a CIA model and investigated the effect of quercetin on NF-κB activity and on the degradation of chondrocyte matrix and apoptosis in rats with RA^57^ and showed that quercetin could protect cartilage by inhibiting NF-κB activation, reducing Matrix metalloproteinase 13 (MMP-13) production, matrix degradation and apoptosis in chondrocytes under inflammatory environment. Jia *et al.* confirmed that quercetin could significantly relieve the arthritis index and paw swelling of CIA mice^58^ and they also reported that quercetin could effectively reduce the expression of inflammatory factors and matrix metalloproteinases in rheumatoid arthritis fibroblast-like synoviocytes (RAFLS).^59^

#### 2.3.3 Effect on zymosan induced RA models

The intra-articular administration of zymosan is an experimental model that promotes inflammatory parameters resembling RA. Guazelli *et al.* indicated that treatment with quercetin dose-dependently could reduce zymosan-induced hyperalgesia, articular edema and the recruitment of neutrophils to the knee joint cavity.^60^

#### 2.3.4 Other effects

Quercetin has also been reported to inhibit the activity of vascular endothelial growth factor (VEGF), basic fibroblast growth factor (bFGF), matrix metalloproteinase-2 (MMP-2) and other cytokines, inhibit angiogenesis and synovial pannus formation. It is suggested that quercetin, plays a role in counteracting RA inflammation, and thus may be reasonably proposed as an adjuvant drug for RA treatment.^61^ Xiao *et al.* collected synovial tissue samples from patients with RA and proved that quercetin can affect the apoptosis of RAFLS by regulating the expression of B-cell lymphoma 2 (Bcl-2) and Bcl-2-associated X protein (Bax). With increasing quercetin concentration, the Bcl-2/Bax value decreases correspondingly, and the apoptosis rate of RAFLS increases.^62^ Furthermore, it has been reported that quercetin can prevent boss loss, so has great potential to be used as a bone health supplement.^63,64^ Quercetin is a potential bioavailability enhancer that could improve the bioavailability of other anti-rheumatoid drugs and play a collaborative role.^65^ There are also some other studies demonstrating the anti-rheumatoid effect of medicinal plant extracts with quercetin as the main component.^62,66–71^ The anti-rheumatoid effect of quercetin has been confirmed and the overall research is summarised in Table 1.

**Table 1:** A summary of studies on quercetin anti-rheumatoid effects and mechanism of action^a^

Effects	Model inducer	Animal	AM	Dose	SW	PT	WT	PWTL	PWMT	TNF-α	IL-1β	IL-1α	IL-6	IL-8	IL-10	IL-17	NO	CRP	MCP-1	PGE2	MMP-1	MMP-3	MMP-13	Ref.
Anti-inflammatory effects	LPS	BALB/c mice	i.p.	0.15 μmol (each mouse)	—	—	—	—	—	↑	↓	—	↑	—	↑	—	—	—	—	—	—	—	—	27
Anti-inflammatory effects	Sodium urate	SD rats	i.g.	100, 200, 400 mg kg^−1^	↓	—	—	—	—	—	—	—	—	—	—	—	—	—	—	—	—	—	—	28
Anti-inflammatory effects	Xylene	BALB/c mice	i.g.	100, 200, 400 mg kg^−1^	↓	—	—	—	—	—	—	—	—	—	—	—	—	—	—	—	—	—	—	28
Anti-inflammatory effects	LPS	*In vitro*	—	2.5, 5, 10 μg mL^−1^	—	—	—	—	—	↓	↓	—	↓	—	↓	—	↓	—	—	—	—	—	—	29
Anti-inflammatory effects	LPS	*In vitro*	—	5, 15, 25 μM	—	—	—	—	—	↓	↓	—	↓	—	—	—	↓	—	—	—	—	—	—	32
Anti-inflammatory effects	PMA	*In vitro*	—	20 μM	—	—	—	—	—	↓	—	—	—	↓	—	—	—	—	—	—	—	—	—	33
Analgesic effects	Hot plate	BALB/c mice	i.g.	100, 200, 400 mg kg^−1^	—	↑	—	—	—	—	—	—	—	—	—	—	—	—	—	—	—	—	—	28
Analgesic effects	Acetic acid	BALB/c mice	i.g.	100, 200, 400 mg kg^−1^	—	—	↓	—	—	—	—	—	—	—	—	—	—	—	—	—	—	—	—	28
Analgesic effects	Bee venom	SD rats	i.g.	40, 80, 120 mg kg^−1^	↓	—	—	↑	↑	—	—	—	—	—	—	—	—	—	—	—	—	—	—	37
Anti-rheumatic effect	Adjuvant-carrageenan	Wistar rats	i.g.	80 mg kg^−1^	↓	—	—	—	—	—	—	—	—	—	—	—	—	—	—	—	—	—	—	52
Anti-rheumatic effect	LPS	—	—	—	—	—	—	—	—	↓	—	—	↓	—	—	—	—	—	—	—	—	—	—	30
Anti-rheumatic effect	AA	Lewis rats	i.g.	150 mg kg^−1^	↓	—	—	—	—	—	↓	—	—	—	—	—	—	↓	↓	—	—	—	—	54
Anti-rheumatic effect	CIA	C57BL/6 mice	i.g.	30 mg kg^−1^	↓	—	—	—	—	↓	↓	↓	↓	—	—	↓	—	—	↓	—	—	—	—	55
Anti-rheumatic effect	CIA	Wistar rats	i.g.	150 mg kg^−1^	↓	—	—	—	—	↓	↓	—	↓	—	—	↓	—	—	—	↓	—	—	—	56
Anti-rheumatic effect	CIA	Wistar rats	i.p.	50 mg kg^−1^	↓	—	—	—	—	—	↓	—	—	—	—	—	—	—	—	—	—	—	↑	57
Anti-rheumatic effect	CIA	DBA/1 mice	i.g.	50 mg kg^−1^	↓	—	—	—	—	—	—	—	—	—	—	—	—	—	—	—	—	—	—	58
Anti-rheumatic effect	TNF-α	*In vitro*	—	50, 100 μM	—	—	—	—	—	—	↓	—	↓	↓	—	—	—	—	—	—	↓	↓	↓	59
Anti-rheumatic effect	Zymosan	Swiss mice		100 mg kg^−1^	↓	↓	—	—	—	↓	↓	—	—	—	—	—	—	—	—	—	—	—	—	108

a Note: ① AM(administration mode), SW (swelling), PT (pain threshold), WT (writhing times), PWTL (paw withdraw thermal latency), PWMT (paw withdrawal mechanical threshold), IFA (incomplete Freund's adjuvant), CRP(C-reactive protein), MCP-1 (monocyte chemotactic protein-1), PMA (phorbol-12-myristate-13-acetate), Ref. (References). ② — not mentioned, ↑ increase, ↓ reduce.

### 2.4 Quercetin *in vivo* metabolism

Metabolism of absorbed flavonoids including quercetin involves their conjugation with glucuronide, sulfate and/or to a limited extent, methylation of the catechol group.^72^ Glucuronidation requires uridine diphosphate glucuronosyltransferase (UDP-GT) and sulfation is dependent on sulfotransferase activity. In general, the major metabolites of quercetin have less bioactivity in comparison to the aglycone, however, there are exceptions in that metabolites may have greater effects *e.g.* the inhibitor constant *K*_i_ for the inhibition of xanthine oxidase by quercetin glucuronides followed the order 4′- > 3′- > 7- > 3-, with quercetin-4′-glucuronide a particularly potent inhibitor (*K*_i_ = 0.25 μM; quercetin *K*_i_ = 0.2 μM).^72^ Quercetin 3-glucuronide, and 3′-methylquercetin 3-glucuronide from 0.1–1 μM inhibited cyclooxygenase-2 (COX-2) expression in lymphocytes *ex vivo* in a dose-dependent manner. However, a single high dose of quercetin (4 μM) does not change COX-2 mRNA expression in human lymphocytes *in vivo*.^73^ To date, a search on flavonoids/quercetin and Arthritis (Rheumatoid) in www.clinicaltrils.gov showed no results.

## 3 Promotion of quercetin pharmaceutical application by nano formulation

Quercetin, as a potential anti rheumatoid drug, is of increasing interest to the pharmaceutical industry. However, its low hydrophilicity and lipophilicity limits its application. Furthermore, it is easily oxidised, and is sensitive to light and temperature. In order to increase its solubility and bioavailability, quite a few researches have been carried out.^74–76^ Among them, the application of nano technology provides promise for the further development and utilization of quercetin.^77^ Nanobiotechnology has been recently regarded as a strategy to improve therapy efficacy by promoting the accumulation of hydrophobic bioactive compounds in tissues.^78,79^ In view of the current research progress, nano formulation of quercetin can improve its solubility, and enhance its bioavailability. At the same time, quercetin nanoparticles can also change the way it is used in medication, control its release rate, and reduce its side effects.

### 3.1 Nano formulation strategies

There has been an increasing focus on the drug delivery potential of nano-formulations in the recent years.^80–84^ Application of nano formulation strategies on bioactive molecules could increase their solubility, absorption, bioavailability, protect them from degradation, prolong their circulation time in plasma,^82,85^ and make them selective biodistribution in the inflammatory parts.^86^ In addition, the nano formulation strategies could also improve intracellular penetration, reduce systemic toxicity and open up the potential for co-delivery of therapeutic agents.^87–89^ Currently, many nano formulated systems designed for therapeutic use of phytochemicals have reached the clinical trial stage and are increasingly applied in clinical practice.^90,91^ So far, a wide variety of nano formulation systems have been used for pharmaceutical applications of quercetin. There are organic^92,93^ and inorganic nanoparticles,^94,95^ nanomicelles,^96,97^ nanoliposomes,^78^ nanoemulsions^98^ and nanocapsules^99^ all gathering great interest. Nanoparticles for delivery of quercetin are generally with a diameter of 20–200 nm. They are formulated using different organic and inorganic materials including dietary fibre,^92^ lactoglobulin,^93^ zein,^100^ casein,^101^ silica,^94^ and quantum dots.^102^ Nanomicelles are self-assembling nanosized colloidal dispersions consist of a hydrophobic core and hydrophilic shell.^103^ Amphiphilic materials are used to synthesise nanomicelles for solubilising hydrophobic biomolecules like quercetin. Nanoemulsions are nanosized emulsions in the range of 20–200 nm, which prepared by either chemical or mechanical methods using mixtures of immiscible liquids, such as water and oil.^104^ Nanoliposomes represent nanosized self-assembled lipid vesicles with a structure of phospholipid bilayers entrapping one or more therapeutic agents.^105^ Detailed applications of different nano-formulations of quercetin on RA treatment are described and discussed in the following sections.

### 3.2 Improvement of solubility

Kakran *et al.* prepared quercetin nanoparticles by evaporation and precipitation nano-suspension (EPN). They studied the type of antisolvent (*e.g.*, water), the effect of concentration and the ratio of solvent to antisolvent of quercetin particles formed in the EPN process. It showed that the solid dispersion significantly improved quercetin solubility.^106^ According to the experiment, quercetin showed a very low dissolution rate with only 10% dissolved within 120 min. For the quercetin nanoparticles, the dissolution rate improved significantly to about 75% at 120 min. It can also be observed that relative dissolution (RD) for quercetin nanoparticles was 7.69 at 120 min, and the time for 50% dissolution was only 7.9 min compared to more than 120 min for quercetin. Moreover, they reported that the size of quercetin nanoparticles was affected by drug concentration, solvent to anti-solvent (S/AS) ratio, stirring speed and flow rate.^107^ The results indicated that the dissolution of quercetin nanoparticles was significantly higher compared with quercetin in simulated intestinal fluid.

In the studies of Khor *et al.*, quercetin was co-precipitated with dietary fibres into a fast-dissolving nano formulation *via* antisolvent precipitation. It was found that a high dissolution rate and good storage stability was achieved for quercetin nano formulations with cellulose fibre, resistant starch, or resistant maltodextrin. The nano formulations exhibited higher levels of antioxidant activities in contrast to quercetin alone.^92^

García-Casas *et al.* reported that a supercritical antisolvent (SAS) process had been used to precipitate microparticles of quercetin together with nanoparticles of cellulose acetate phthalate (CAP). Release profiles of quercetin were carried out in simulated gastric and intestinal fluids. Higher ratios of quercetin to polymer in the coprecipitates were recommended to achieve faster release and higher solubilities of quercetin.^109^

Wang *et al.* reported that amphiphilic chitosan was obtained through grafting of deoxycholic acid modified chitosan and *N*-acetyl-l-cysteine. Quercetin-loaded nanomicelles (CS-DA-NAC-QNMs) were prepared through a self-assembly method by using amphiphilic chitosan as the wall-material and quercetin as core-material. They demonstrated that there was a bursting release of quercetin from CS-DA-NAC-QNMs for 0 to 8 hours, and then the release rate decreased gradually. After 72 hours, more than 40% of quercetin were released. All the quercetin loaded nano micelles samples showed good hemocompatibility, and their water solubility and biocompatibility was increased significantly.^110^

Chavoshpour-Natanzi *et al.* prepared β-Lactoglobulin (BLG) nanoparticles for the encapsulation of quercetin. The nanoparticles had a mean particle size of between 180–300 nm and a loading efficiency (LE) of 13.9%. Protein nanoparticles could be digested at different stages of the gastrointestinal tract, depending on several factors including specificity of proteases *e.g.* pepsin. This study suggested that nano formulation could overcome BLG resistance to peptic digestion. Thus synthesised BLG-quercetin nanoparticles could achieve controlled release of quercetin under simulated conditions.^93^

The study performed by Simon *et al.* used harmless amphiphilic polyoxazolines (POx) to encapsulate quercetin.^96^ They produced mixed micelles, made of POx and phosphatidylcholine, using a thin film and high-pressure homogeniser process. The obtained nanomicelles that were about 20 nm in diameter with a spherical shape and encapsulation efficiency of 94 ± 4%. They demonstrated improved cell viability and antioxidant activity from these nanomicelles compared to quercetin alone. Subsequently, this group synthesised quercetin encapsulated lipid nanocapsules (LNC) using the same material POx.^111^ A similar synthesis method has been used as for the production of mixed micelles but implementing an additional short sonication step. The obtained LNC have a well-defined spherical shape and a size of ∼30 nm.

### 3.3 Enhancement of bioavailability

Jeyadevi *et al.* investigated the anti-arthritic activity of quercetin with thioglycolic acid capped cadmium telluride quantum dots (TGA-CdTe QDs) as nano carrier on adjuvant induced arthritic Wistar rats.^102^ Fifteen days after adjuvant induction, arthritic rats received QDs-quercetin complex orally at a dose of 0.2 and 0.4 mg kg^−1^ daily for 3 weeks. The complex induced a significant reduction in inflammation and improvement in cartilage regeneration.

Aditya *et al.* reported a comparative study of solid lipid nanoparticles (SLN), nanostructured lipid carriers (NLC), and lipid nano emulsions (LNE) of quercetin.^112^ Encapsulation efficiency (EE) of quercetin in these nanocarriers was above 90%. Maximum bio-accessibility was observed with NLC and LNE (W60%) compared to SLN (W35%) and free quercetin solution (W7%).

Tran *et al.* developed a quercetin-containing self-nanoemulsifying drug delivery system (Q-SNEDDS). Oil-in-water nanoemulsions were formed to improve quercetin oral bioavailability.^98^ Following oral administration of Q-SNEDDS in rats (15 mg kg^−1^), the maximum concentration (*C*_max_) of plasma quercetin after 24 h was 3.75 ± 0.96 mg L^−1^, increased by approximately three-fold compared with the native quercetin group (1.20 ± 0.17 mg L^−1^). The results suggested that Q-SNEDDS can enhance the solubility and oral bioavailability of quercetin. Collectively, Q-SNEDDS increased quercetin *C*_max_ and area under the concentration curve (AUC), from 6.7 ± 1.4 L^−1^ h^−1^ to 14.0 ± 2.8 L^−1^ h^−1^, without affecting its elimination kinetics, suggesting that Q-SNEDDS improved quercetin bioavailability by enhancing its absorption.

Dinesh Kumar *et al.* have also studied biodegradable polymeric nanoparticles for the effective delivery of quercetin. The results suggest that optimised formulation of nanoparticles could promote the controlled release and improve the physical stability of quercetin.^113^

Lee *et al.* investigated the antioxidative and anti-inflammatory activities of quercetin-loaded silica nanoparticles (QLSNs).^94^ QLSNs were synthesised using an oil-in-water microemulsion method. The nanoparticles showed comparable cell viability to that of the free quercetin, while the amounts of proinflammatory cytokines produced by macrophages, such as TNF-κB, IL-6, and IL-1β, were significantly reduced.

Caddeo *et al.* prepared cross-linked chitosan liposomes of quercetin and confirmed that the system had acid resistance and promoted the release under alkaline conditions.^114^ In addition, Hao *et al.* proposed a facile electrostatic deposition method to prepare quercetin nanoliposomes coated with chitosan.^115^ The obtained Q-NPs have high EE (71.14%) and the storage stability and antioxidant activity was improved compared with native quercetin.

Penalva *et al.* studied the use of zein nanoparticles as a carrier for the oral delivery of quercetin. Quercetin and 2-hydroxypropyl-β-cyclodextrin were encapsulated together in zein nanoparticles. They showed that nanoparticles provided high and sustained levels of quercetin in plasma after oral administration. The *C*_max_ of plasma quercetin was 176 ± 13.4 μg mL^−1^. The mean values obtained for AUC and the half-life of the terminal phase (*t*_1/2_) were 167 ± 8.21 μg h mL^−1^ and 0.60 ± 0.35 h, respectively. The mean residence time (MRT) was 1.60 ± 0.12 h, whereas the quercetin clearance and its volume of distribution were calculated to be 30 mL h^−1^ and 26 mL h^−1^, respectively. The relative oral bioavailability was calculated to be about 60%.^116^ They further optimised the preparative process of quercetin loaded casein nanoparticles and evaluated the pharmacokinetics of the nanoparticles after oral administration to Wistar rats^117^ showing that the relative oral bioavailability of quercetin in nanoparticles (close to 37%) was about 9-times higher than the oral solution of quercetin in a mixture of PEG 400 and water. Another study by Li *et al.* also used zein and soluble soybean polysaccharide (SSPS) nanoparticles. The EE of quercetin was greatly improved to 82.5% and the photochemical stability and 2,2′-azino-bis(3-ethylbenzothiazoline-6-sulfonic acid (ABTS^+^) scavenging ability of quercetin in such nanoparticles were significantly enhanced.^100^

Pivetta *et al.* produced nanostructured lipid carriers to load quercetin. Their results indicated that the nanoparticles exhibited a low recrystallization index (13.03%) which is important to obtain high entrapment efficiency (97.42%) and avoid drug expulsion during the storage time.^118^ Furthermore, in a reconstructed human skin model, it was observed that the topical formulation of quercetin-NLC presented no phototoxic potential. Therefore, this developed nanostructure is a vehicle with potential for topical administration of quercetin.

Research by Gokhale *et al.* reported a quercetin loaded nano emulsion (NE)-based gel for the effective of management RA.^119^ This study showed that quercetin-NE has no toxic effect on synoviocytes and a strong inhibitory effect on LPS-induced TNF-α production. It has also exhibited adequate rheological behaviour with a good texture profile and improved drug permeation compared to a free quercetin gel. In addition, the gel was found to be non-irritating and inhibited the formation of paw edema in rats induced by Freund's complete adjuvant (CFA) over 24 hours. Another study performed by Ghatak and Iyyaswami used casein particles to encapsulate quercetin to improve its water solubility and bioavailability.^101^ A maximum encapsulation yield of 97% could be achieved with the addition of 0.5% (w/v) sodium caseinate, 0.1 M of calcium chloride, 0.5 M of di potassium hydrogen phosphate, 0.1 mM CTAB and 1 M of sodium citrate at a pH of 7.

### 3.4 Regulation of release rate

Liu *et al.* studied the characterization and biodistribution of quercetin-loaded cationic nanostructured lipid carriers (QR-CNLC) *in vivo*. QR-CNLC nanoparticles were prepared by emulsification at high temperature and subsequent solidification at low temperature.^78^ QR-CNLC exhibited an average particle size of 126.6 nm and 89.3% entrapment efficiency. The results demonstrated that QR-CNLC offered slower release compared with quercetin solution *in vitro*.

Mohan *et al.* reported on TiO_2_ nanotubes that were loaded initially with quercetin (TNTQ) and then additionally with chitosan coated on the top of the quercetin (TNTQC) to various thicknesses. The drug release of TNTQ and TNTQC were studied in Hanks' solution for 192 hours. The results showed that the release of drug into the local environment during that duration was constant and the local concentration of the drug could be controlled and tuned by controlling the thickness of the chitosan (0.6, 1 and 3 μm).^120^

Zong *et al.* studied *in vitro* release of quercetin-loaded mixed micelles composed of Pluronic P123/Poloxamer 188, and their pharmacokinetics in rat.^121^ The results indicated that quercetin-loaded mixed micelles have high entrapment efficiency and loading efficiency which could improve the release behaviour *in vitro*. The nano formulation of quercetin also prolonged the circulation time of quercetin and significantly enhanced the bioavailability of quercetin. Hui *et al.* prepared and characterised amphiphilic chitosan/quercetin nano micelles (ACS-QNMs) using a novel amphiphilic chitosan (ACS).^97^ ACS has deoxycholic acid (DA) as the hydrophobic group and n-acetyl-l-cysteine (NAC) as the hydrophilic group. The results showed that quercetin could be released *in vivo* and was stable when stored at room temperature after being embedded in nano micelles.

Zhao *et al.* prepared a new nanodrug delivery system (quercetin@mesoporous hydroxyapatite, QUE@MHAs) and investigated its release *in vitro*.^122^ The results showed that QUE@MHAs have good stability and a slow drug release rate.

### 3.5 Transdermal administration

Quercetin is a flavonoid with significant antioxidant and anti-inflammatory activities and can be considered as a potential topical drug for skin. Nevertheless, it suffers from poor water solubility and consequently topical inactivity. To enhance its transdermal absorption, a number of different nano formulations of quercetin have been studied, including liposomes, nanoparticles, micelles, and solid lipid nanoparticles.

Tan Qi studied the preparation and evaluation of quercetin-loaded lecithin–chitosan nanoparticles for topical delivery. Quercetin nanoparticles were prepared using organic solvent injection. The results demonstrated that the nanoparticles could clearly increase the amount of drug retention in especially in the epidermis and also in the dermis, and further enhance antioxidation and anti-inflammatory effects.^123^

Guo *et al.* evaluated the potential of quercetin-loaded nanostructured lipid carriers (QT-NLCs) as a topical delivery system.^124^ The nanoparticles were prepared by the method of emulsion evaporation–solidification at low temperature. The results showed that QT-NLCs could promote the permeation of quercetin, increase the amount of quercetin retention in epidermis and dermis, and enhance the effect of anti-oxidation and anti-inflammation exerted by quercetin.

Sapino *et al.* evaluated the potential of aminopropyl functionalised mesoporous silica nanoparticles (NH_2_-MSN) as topical carrier system for quercetin delivery. The silica nanoparticle vehicle prevented UV-induced degradation of quercetin over time, which showed positive effect on photostability of quercetin. Epidermal accumulation and transdermal permeation were evaluated *ex vivo* using porcine skin mounted on Franz diffusion cells. The inclusion complexation with the inorganic nanoparticles increased the penetration of quercetin into the skin after 24 hours post-application without transdermal delivery.^95^

Hatahet *et al.* tested three approaches to improve quercetin delivery to skin, including liposomes, lipid nanocapsules (LNC) and smartCrystals®.^99^ They showed that compared to liposome (0.56 mg mL^−1^), quercetin smartCrystals® and LNC had a better drug loading with 14.4 mg mL^−1^ and 10.8 mg mL^−1^ respectively. SmartCrystals® and LNC demonstrated different skin penetration behaviours. Only LNC allow quercetin to be delivered to viable epidermis that holds potential for treatment of skin inflammatory disorders.

In conclusion, quercetin is not only an important drug source for oral administration but can also be used as a transdermal absorbent by employing nanotechnology to enhance its transdermal absorption capacity. It can be seen from all above reports that different materials and forms of nano encapsulation can have many positive effects on quercetin anti-rheumatoid applications. Nano formulation has significantly improved the solubility and bioavailability of native quercetin, and at the same time avoiding its shortcomings. The anti-rheumatoid related nano formulations of quercetin are summarised in Table 2.

**Table 2:** A summary of anti-rheumatoid related nano formulation of quercetin^a^

Type	Nanocarrier	Preparation method	Antisolvent	Size (nm)	Aim	Ref.
Nanoparticles	—	Syringe pump	Deionised water	170	Improvement of solubility	107
Nanoparticles	Dietary fiber	Antisolvent precipitation	Water	<100	Improvement of solubility	92
Nanoparticles	Cellulose acetate phthalate	Supercritical antisolvent	Supercritical CO_2_	145	Improvement of solubility	109
Nanoparticles	β-Lactoglobulin	Antisolvent precipitation	Acetone	180–300	Improvement of solubility	93
Nanoparticles	Thioglycolic acid-capped cadmium telluride quantum dots	Antisolvent precipitation	Acetone	185	Enhancement of bioavailability	102
Nanoparticles	Polycaprolactone	Nano-precipitation	Pluronic F127	213–257	Enhancement of bioavailability	113
Nanoparticles	Silica	Oil-in-water microemulsion	Water	70–140	Enhancement of bioavailability	94
Nanoparticles	Zein, 2-hydroxypropyl-β-cyclodextrin	Desolvation	Water	300	Enhancement of bioavailability	116
Nanoparticles	Casein, 2-hydroxypropyl-β-cyclodextrin	Simple coacervation	Water	200	Enhancement of bioavailability	117
Nanoparticles	Natural lipids	Emulsion and sonication	Pluronic F68	130	Enhancement of bioavailability	118
Nanoparticles	Zein, SSPS	Antisolvent precipitation	—	200	Enhancement of bioavailability	100
Nanoparticles	Casein	Emulsion	Ethanol	114.3–482.1	Enhancement of bioavailability	101
Nanoparticles	Mesoporous hydroxyapatite	Magnetic stirring	Deionised water	169–179	Regulation of release rate	122
Nanoparticles	Lecithin, chitosan	Organic solvent injection	Ethanol	95	Changes of administration mode	123
Nanoparticles	Soya lecithin, glyceryl monostearate, stearic acid, media chain triglyceride	Emulsion evaporation–solidification	Water	215	Changes of administration mode	124
Nanoparticles	Mesoporous silica	Impregnation and magnetic stirring	Methanol	200–300	Changes of administration mode	95
Nanomicelles	Amphiphilic chitosan	Grafting deoxycholic acid, *N*-acetyl-l-cysteine	—	360–580	Improvement of solubility	110
Nanomicelles	Polyoxazolines, phosphatidylcholine	Thin film and high pressure homogeniser	Acetonitrile	20	Improvement of solubility	96
Nanomicelles	Polyoxazolines, Labrafac®, Lipoid® S75	Thin film and high pressure homogeniser process	Acetonitrile	30	Improvement of solubility	111
Nanomicelles	Pluronic P123/Poloxamer 188	Film dispersion	Tween80	—	Regulation of release rate	121
Nanomicelles	Amphiphilic chitosan	Self assembly	Deionised water	140–600	Regulation of release rate	97
Nanoliposomes	Cross-linked chitosan	Ultrasonication	TPP aqueous solution	180	Enhancement of bioavailability	114
Nanoliposomes	Chitosan	Facile electrostatic deposition	Chloroform, methanol	350–600	Enhancement of bioavailability	115
Nanoliposomes	GMS, MCT, soy lecithin	Emulsifying and solidifying	Transcutol	118–135	Regulation of release rate	78
Nanoliposomes	DPPC, Cremophor® EL	Magnetic stirring	Alcohol	179	Changes of administration mode	99
Nanoemulsions	Lecithin	High pressure homogeniser	—	73–91	Enhancement of bioavailability	112
Nanoemulsions	Self-nanoemulsifying drug delivery system	Gentle stirring	Tween 80, PEG 400	204–213	Enhancement of bioavailability	98
Nanoemulsion-based gels	Arachis oil, oleic acid	Gentle stirring	Tween 20, PEG 400	137	Enhancement of bioavailability	119
Nanosuspensions	—	Evaporative precipitation	Hexane	220	Improvement of solubility	106
Nanotubes	TiO_2_	Top filling	—	125 (tubes diameters)	Regulation of release rate	120
Nanocapsules	Lipid	Magnetic stirring	Milli Q water	26	Changes of administration mode	99

a Note: — not mentioned; Ref. (reference).

## 4 Conclusions and future perspective

Rheumatoid arthritis is a common worldwide public health problem.^125^ It is in the top ten of the world's disease spectrum and is also one of the diseases that seriously affect human physical and mental health, with high rates of disability and death. The pathogenesis of RA is very complex and has not been fully elucidated. RA can be managed through conventional treatments, but more attention has recently been paid to the treatment of RA by natural active ingredients from medicinal plants, including food plants, because of their safety and efficacy. In recent years, the consumption of plant-based medicines and other botanicals has increased. According to an estimate of World Health Organization (WHO), nearly 80% of the populations of developing countries rely on traditional medicines.^126^

As a plant derived medicine, quercetin is a striking candidate for use in arthritic therapy.^126^ As summarised above, there are many reports on the effects of quercetin on RA. It has been confirmed that quercetin has significant anti-inflammatory and analgesic effects *in vivo* and *in vitro* and furthermore, quercetin and its derivatives also have significant antioxidant effects, which is one of the possible reasons for their significant anti-rheumatic properties.^20^ At the same time, it is reported that quercetin is mostly well tolerated and safe to use. Doses up to 1000 mg each day for several months have not produced adverse effects on blood parameters, and liver, and kidney function. As a potential bioavailability enhancer for active pharmaceutical ingredients, quercetin can also be used as one of the options in combination therapy for RA.^65^ Moreover, it has been shown that with the application of nano formulations, quercetin has not only improved oral bioavailability, but also can be used for external transdermal use, which provides a new reference for the treatment of RA.

Move rover, Susanne Andres *et al.* reviewed the safety aspects of quercetin as a dietary supplement. It showed that based on animal studies, some possible critical safety aspects of quercetin could be identified such as to enhance nephrotoxic effects in the predamaged kidney or to promote tumour development especially in estrogen-dependent cancer.^127^ Furthermore, when quercetin interacts with some drugs, the bioavailability of may be altered. Therefore, it suggests that, like any potential drug or active ingredient, a very in-depth study on its safety and applicability should be conducted before clinical application. Future clinical studies are needed to verify the safety and efficacy of nano formulated quercetin as a new RA treatment medicine. Future clinical studies are needed to verify the safety and efficacy of Nano formulated quercetin as a new RA treatment medicine.

## Conflicts of interest

There are no conflicts to declare.

## Supplementary Material
